# The Role of Companion Animals in the Transition of Care: A Case Report

**DOI:** 10.7759/cureus.74094

**Published:** 2024-11-20

**Authors:** Juan Gabriel Jimenez Garcia, Juan Ramon Santos Rivera, Guillermo Izquierdo-Pretel

**Affiliations:** 1 Internal Medicine, Florida International University, Miami, USA; 2 Internal Medicine, Ponce Health Sciences University, Ponce, PRI

**Keywords:** barriers of care, companion animals, healthcare disparities, homelessness, patient discharge, transition of care

## Abstract

Numerous individuals experiencing homelessness have a pet. When a homeless person is hospitalized for an emergency medical need, discharge planners are sometimes faced with tough options and a lack of resources for safe discharges from the hospital. We detail the case of a 64-year-old female patient who was admitted due to a witnessed syncopal event. The patient was admitted with her companion dog, which remained at the bedside through her hospitalization. The workup for her syncopal event was negative (CT brain, carotid US, ECG, troponins, orthostatic). Her discharge process was complicated by her need for a shelter placement that would accommodate her and her pet dog. The patient was discharged without finding a shelter that could accept her dog, which prevented her from receiving home health physical therapy. This case illustrates how pet ownership can create additional barriers to the transition of care for homeless individuals, limiting access to necessary follow-up services and impacting overall recovery outcomes. Thus, we would like to highlight this barrier that exists in this vulnerable population.

## Introduction

Homelessness is a multifactorial problem that refers to people living in shelters or crisis accommodation, living on the streets or in vehicles, and staying temporarily with friends and family. In the year 2023, approximately 653,000 people were homeless, out of which only 40% of them lived in shelters [1]. Homeless people are more vulnerable to chronic conditions due to a variety of factors like stigma and lack of health insurance. Their diseases and chronic conditions often go untreated until they require hospitalization. This has a direct impact on their life expectancy, reducing it by 8-22 years when compared to those who are not homeless [2,3].

Approximately 5-25% of the homeless population in the USA report having a pet dog or cat [4-6]. Furthermore, older adults experiencing loneliness or mental health issues reported being more likely to have a pet as a companion [7]. Homeless youth with pets reported experiencing significantly less loneliness and fewer symptoms of depression compared to those without pets [8]. This highlights the association between pet ownership and emotional support within the homeless population, underscoring the important role pets play in their well-being. Unfortunately, since most shelters and other supportive facilities do not allow animals, owning a pet while homeless creates an extra obstacle to accessing this aid [2,8,9]. Homeless people develop profound relationships with their pets, and they end up regarding their pets as family [10]. Because of this, they may choose to stay with their pet and not receive any service that may require them to abandon their pet. In this case, we describe a patient who had an elevated risk of readmission from her comorbidities, and her illness required shelter placement and health assistance. However, she was unable to be placed in a shelter due to her pet dog.

## Case presentation

**Figure 1:**
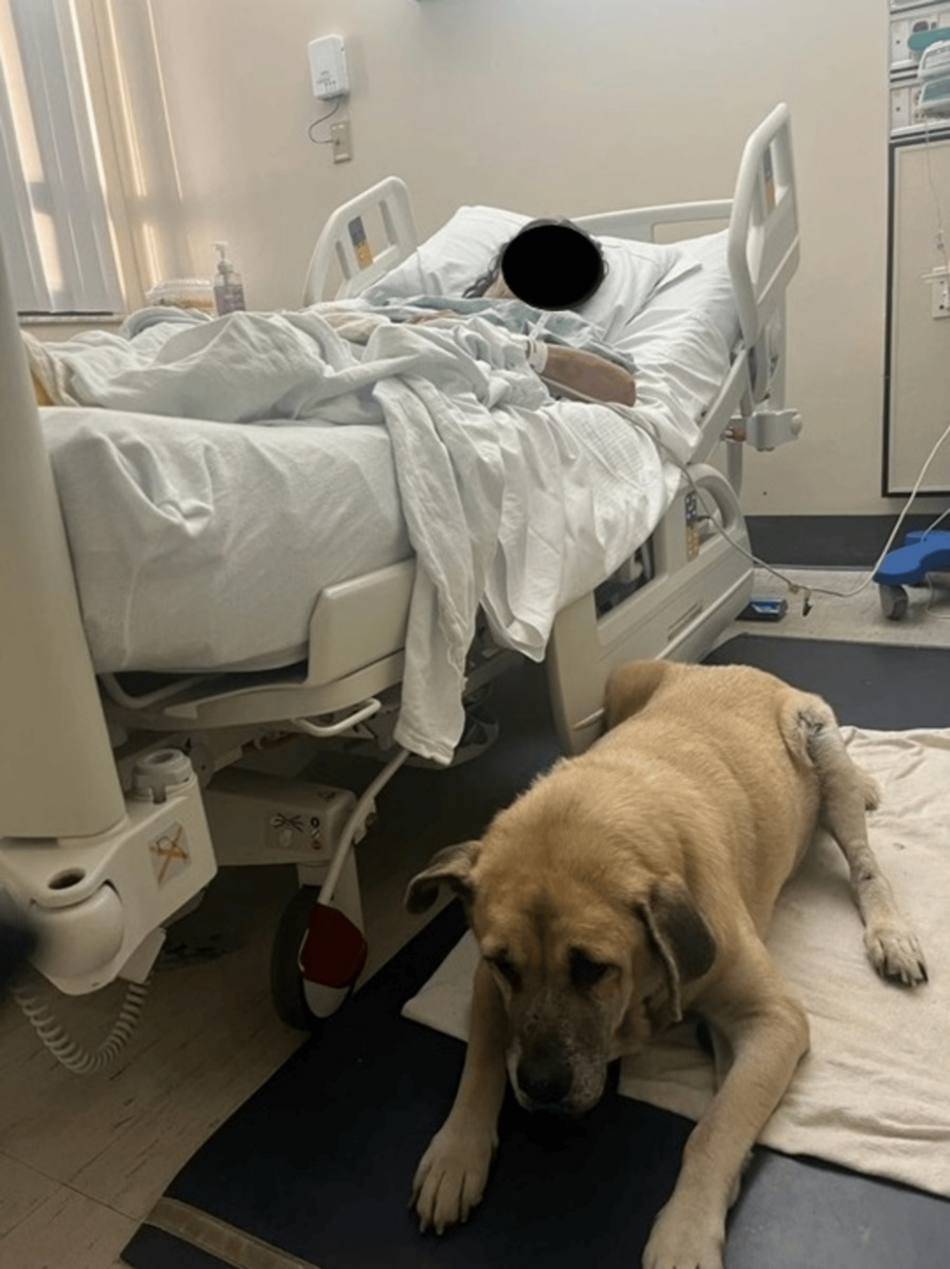
The patient, with her face concealed to maintain anonymity, is pictured at bedside with her pet dog. Verbal consent was obtained from the patient for capturing and using this image in the case report.

A 68-year-old female patient with a complex history of homelessness, hypertension, hyperlipidemia, diabetes mellitus type II, and coronary artery disease with percutaneous coronary artery intervention with three stent placements in 2017 and 2019; a history of cerebrovascular accident in 2017; and an active tobacco smoker who presented to the hospital after a witnessed syncopal event. The patient does not recall the event. However, she reported having previous syncopal events, though no such episodes were documented in our hospital records. Additionally, she stated being unable to keep to a proper diet or with her scheduled medications due to her homelessness status. During hospitalization, she requested her companion dog to accompany her at the bedside (Figure 1).

The physical exam was negative for abnormal auscultation of her heart or lungs. For musculoskeletal, she ambulates with a rollator, and she has bilateral 3+/5 hip strength, 2/5 hip extension, 3/5 knee flexion, 3/5 knee extension, 2+/5 dorsiflexion, and 3-/5 plantar flexion. On her right hand, there was the presence of Dupuytren's contracture of the fourth finger. She did have mild erythematous patches on bilateral inferior skin folds of the breasts, with red plaques and lichenification in the mid-sternum.

For her work-up, a chest X-ray was ordered, which was unremarkable. Additionally, a bilateral carotid ultrasound and duplex were done, which showed no significant carotid stenosis. An echocardiogram obtained a month prior showed an ejection fraction of 55-60% and mild aortic sclerosis, otherwise unremarkable. A CT of the brain without contrast was negative for hemorrhage, mass effect, or any acute pathology. The patient refused the stress test as she did not want her dog to leave her side. It was determined at this time that the likely cause of the syncopal event was most likely to be secondary to hypoglycemia and hypovolemia, as she had poor hydration and alimentation. She remained stable during hospitalization with no neurological deficits, presyncope, or syncopal events.

During the hospitalization, physical therapy is recommended for the patient to receive home health physical therapy for strengthening and to improve her performance status. However, the patient had a significant barrier to transition due to her homelessness status and service/companion dog, albeit having Medicare. The social workers operated on finding placement for the patient, as she posed an elevated risk of readmission if discharged to the streets. However, the social workers were unable to find shelter placement in the county, yet they did find a skilled nursing facility that would accommodate her with her dog. Still, the patient refused as it was in another county. The patient was discharged to herself and left the hospital with her companion dog.

## Discussion

For around 25% of the homeless population, pets are a significant source of loving relationships for those experiencing this crisis [8,10]. It is crucial to maintain the pet owners' connection to their animals since the social support of human-animal ties is crucial for this group because it offers structure and routine both during and after their crises [10]. However, since there are few pet-friendly laws, this vulnerable group frequently suffers difficulties finding short-term shelter and long-term, reasonably priced housing, or having to leave their pet [2]. Furthermore, these pet owners may have less access to services after hospitalizations if they are unable to leave their dogs or cats safely.

The majority of homeless patients hospitalized are not questioned about their housing status during their hospital stay and those who have often encountered shortcomings in coordination and communication between healthcare providers and shelters, leading to discharges to shelters where patients often are turned away due to late discharge timings [11,12].

Homeless people are frequently faced with the difficult decision of giving up their valued pets to get more resources, but many firmly refuse to do so since they view their pets as part of their family [6]. There is a significantly limited number of studies on the topic of pet ownership among the homeless population and how it poses a major obstacle to their transitioning out of inpatient care [2,4,13]. More preparation and improved lines of communication are undoubtedly required during the discharge process to determine what constitutes a suitable discharge for those who are homeless [9]. Evidence demonstrating the link between ill health and homelessness should motivate health institutions to advocate for placement solutions [7]. Housing services and shelters should offer pet-friendly accommodations, such as outdoor and indoor housing options for pets, designated pet-friendly areas or floors, and support services like free veterinary care and pet food [14]. It is essential to consider all potential obstacles that a homeless patient might face upon discharge, including their companion animal, which is frequently overlooked in this context.

## Conclusions

Homeless patients face various challenges when discharged, particularly those who are older or have a pet or service animal. Often when there is a need for placement to a shelter, assisted living facility, or skilled nursing facility, this is limited due to not having pet-friendly options. Homeless individuals with pets are reluctant to separate from their animal companions because they see them as integral family members above any potential gains they might obtain. Therefore, this article aims to call attention to what housing services ought to do, which is to cater to pet-friendly alternatives. Nonetheless, this may pose a challenge at first, yet the overall goal of caretaking for an individual with necessities should be the standard. The case presented highlights the unique limitation faced by the homeless population that has a pet or service animal and their burden to gain access to a shelter placement and possibly other types of health assistance. Our goal is to bring awareness to this unique vulnerability that can be encountered but is not frequently studied.
